# Satisfaction with Care Received at the End of Life in Portugal: A Systematic Review

**DOI:** 10.3390/nursrep15060221

**Published:** 2025-06-18

**Authors:** Amélia Ferreira, Alexandra Pereira, Sara Pinto

**Affiliations:** 1 School of Medicine and Biomedical Sciences of Abel Salazar Biomedical Institute, R. Jorge de Viterbo Ferreira 228, 4050-313 Porto, Portugal; amnpereira@ulsts.min-saude.pt; 2 Community Care Unit of Lousada, Rua de Santo Tirso 70, 4620-848 Lousada, Portugal; 3 RISE-Health, Nursing School of Porto, Rua Dr. António Bernardino de Almeida 830/844/856, 4200-072 Porto, Portugal; sarapinto@esenf.pt; 4 Centre for Innovative Biomedicine and Biotechnology (CIBB), University of Coimbra, Rua Larga, 3004-504 Coimbra, Portugal

**Keywords:** caregivers, delivery of healthcare, end-of-life care, nursing, palliative care, patient satisfaction, Portugal, systematic review

## Abstract

**Background**: Although there is recent evidence from a range of sources that demonstrates the usefulness of assessing satisfaction with healthcare, there is still a lack of evidence regarding the satisfaction with healthcare received by adult individuals at the EOL in Portugal. **Methods**: This systematic review aims to examine and synthesize the evidence published through 2024 regarding satisfaction with care received by adult individuals at the EOL in Portugal. **Results**: Seven studies were included in this review. Six out of the seven studies were quantitative descriptive cross-sectional studies. The majority of them used family caregivers as the source of information. Most studies had small samples that characterized the satisfaction with care received in a palliative care context in a specific region of the country. Sixteen different variables were studied to understand the factors that influence satisfaction with care. The majority of them were related to the personal characteristics of the patient or the family caregiver. No evidence was found regarding the study of variables related to the patient’s trajectory, location of care provision, or place of death. **Conclusions**: The different instruments used in the data collection in all of the included studies made it difficult to compare the results and draw definite conclusions regarding satisfaction with care received by adult individuals at the EOL in Portugal. Although different levels of satisfaction with the care were found, it can be concluded that the results tend to be positive in the palliative care context.

## 1. Introduction

The demographic transitions observed worldwide in recent decades have escalated the complexity and extent of healthcare needs. Characterized by increased longevity and the rising prevalence of chronic diseases, this global context has intensified the demand for comprehensive care [1,2]. Nearly 69% of palliative care requirements are associated with non-communicable diseases [3]. Projections suggest that by 2060, the levels of serious health-related suffering may nearly double, particularly among older adults [4]. This situation has prompted a significant reorientation toward the integration of palliative care across various settings, moving away from merely prognosis-based access [5,6]. Patients and their families increasingly express a preference for receiving end-of-life (EOL) care at home [7], even though a majority of deaths occur in institutional environments [8].

This urgent need to adapt healthcare systems is particularly noticeable in Portugal, a country marked by a high life expectancy [9], a considerable prevalence of palliative care needs [3], and serious health-related suffering [10]. Over half of the Portuguese population prefers to die at home [8], yet more than 60% of deaths take place in hospitals [11], highlighting a pressing need to reformulate palliative care policies.

Portugal is recognized for its advanced integration of palliative care services within a national framework, which includes inpatient units, hospital-based teams, and community-based support [3]. However, access to these services remains uneven, with resources concentrated in coastal and urban regions [12], resulting in many individuals receiving palliative care in non-specialized settings [13].

The increasing healthcare needs and growing demand for resources at the EOL highlight the need to redefine evidence-based measures that allow the evaluation of care quality [14,15]. In the EOL context, the quality of care focuses on optimizing healthcare delivery according to both an individual and a societal perspective [16]. However, satisfaction with care assesses the fulfillment of individual needs, as well as patients’ and families’ expectations and preferences [17].

While systematically assessing patient and family needs and preferences is paramount, satisfaction with care stands as a widely recognized and important outcome in palliative care that should never be overlooked, as it offers invaluable insights into the patient and family experience [18,19,20]. Within the context of end-of-life care, assessing satisfaction takes on critical importance across several dimensions. Beyond its contribution to the continuous improvement of healthcare delivery and organization, it significantly enhances patient and family involvement in care decisions. This active participation fosters a profound sense of recognition of their human dignity, directly leading to an improved quality of life during a profoundly challenging period. In many ways, the systematic evaluation of satisfaction at life’s end directly contributes to realizing the very essence of palliative care itself [21]. Furthermore, satisfaction assessment often extends to bereaved family members. Their participation provides a crucial sense of closure, helping to make the end-of-life experience more humane and positive, and ultimately supporting a more effective grieving process [22]. Recent evidence highlights that enhancing family members’ satisfaction with care has the potential to improve their post-loss quality of life [23].

In parallel with the increasing global attention to patient satisfaction as a measure of care quality, Portugal faces the challenge of assessing satisfaction with EOL care, recently defined as an important indicator of palliative care (PC) delivery [24]. This challenge is compounded by the need to adapt and implement effective evaluation systems, particularly within palliative care, where a standardized evaluation tool is absent and the number of validated instruments remains limited [25].

While the utility of assessing satisfaction with care is increasingly recognized [19], the specific context of patient and family satisfaction with EOL care in Portugal remains under-researched, leaving a significant gap in our understanding of patient and family experiences [26]. Assessing satisfaction with end-of-life care in Portugal across all care settings would make it possible to identify and enhance best practices and would constitute a clear contribution to the development and wider implementation of palliative care. To address this and to inform the development of patient-centered palliative care strategies, this systematic review aims to examine and synthesize the evidence regarding satisfaction with care received by adult patients at the EOL in Portugal. Considering this, we formulated the following PICO question: “What is the level of satisfaction with the healthcare received by adults at the end-of-life in Portugal across different care settings?”.

## 2. Materials and Methods

This systematic review followed the Joanna Briggs Institute methodology for systematic reviews [27], and it was reported using the Preferred Reporting Items for Systematic Reviews and Meta-analyses (PRISMA) guidelines [28].

### 2.1. Eligibility Criteria

#### 2.1.1. Type of Participants

This review examined studies focused on patients diagnosed with various life-threatening or terminal illnesses who were in EOL care and their family caregivers. We included studies concerning adult patients (≥18 years) receiving EOL care in Portugal, regardless of their nationality, race, or ethnicity.

EOL patients were defined as those explicitly identified as having a life expectancy of less than one year or those receiving specialized palliative care services (whether in inpatient units, outpatient settings, or community-based care) [29,30]. When discussing family caregivers, we define “family” in a broader sense that goes beyond biological or legal relationships (such as marriage, birth, or adoption) to include other meaningful relations, such as close friends, neighbors, or legal representatives [7]. This inclusive interpretation of family was applied throughout the review.

Studies focusing on individuals with advanced chronic illnesses or life-threatening illnesses who were not identified as being at the EOL or receiving palliative care were excluded. Additionally, we also excluded studies that primarily addressed the perspectives of healthcare professionals, volunteers, or non-family caregivers (such as informal caregivers who receive payment).

#### 2.1.2. Phenomena of Interest

We included studies that focused on satisfaction with EOL care from the perspectives of both patients and their family caregivers, irrespective of the evaluation instruments used. By satisfaction with EOL care, we mean the extent to which patients and/or family caregivers perceived that the care provided meets their needs, expectations, and preferences. This satisfaction can encompass several aspects, including but not limited to symptom management, communication strategies, emotional and spiritual support, respect for patient preferences, and the overall quality of care received [31].

#### 2.1.3. Context

We included studies that involved participants at the EOL in a variety of care settings, including but not limited to specialized palliative care services (inpatient, outpatient, and community care), long-term care facilities, acute care settings, and community-based care.

#### 2.1.4. Type of Studies

We included randomized controlled trials (RCTs), cohort studies, and observational studies, regardless of whether they followed quantitative, qualitative, or mixed-method approaches. Reviews (such as scoping reviews, rapid reviews, and narrative reviews), validation studies of instruments, clinical practice reports, studies based on expert opinion, commentaries, editorials, letters to the editor, and conference abstracts were excluded.

To be eligible for inclusion, studies had to meet the following criteria: (1) a clear description of the study objectives; (2) a clear indication that patients had a prognosis of less than one year of life and were receiving care in Portugal, or that they were receiving specialized palliative care services in Portugal; (3) the use of valid and reliable measures of EOL care satisfaction; and (4) a comprehensive explanation of the methodology and data analysis methods.

### 2.2. Search Strategy

Although this systematic review was not registered prospectively, a comprehensive search was conducted in PROSPERO, and no protocols with the same objectives were found. Therefore, in July 2024, a preliminary search in Medline and CINAHL (via EBSCO) was conducted to explore relevant literature and identify the index terms used to describe the studies. Based on our search, we created a matrix of key terms and developed a comprehensive search strategy, which we then adapted for all relevant sources.

In December 2024, three databases were searched: CINAHL (since 1981), Medline (since 1950), and PsycInfo (since 1806). The search strategy included both controlled and free-text vocabulary, namely ‘end-of-life’, ‘palliative care’, ‘satisfaction’, and ‘Portugal’ (Appendix A). No timeframe filter was applied in our search strategy. This decision was made to ensure the most comprehensive retrieval of relevant studies, given the scarcity of published research on satisfaction with end-of-life care in Portugal and the enduring relevance of older findings in this area.

Additionally, the reference lists of all of the included studies were searched, and relevant authors and investigators were contacted for further studies and unpublished data. A search for unpublished studies was also conducted in IndexRMP (Índex de Revistas Médicas Portuguesas) and RCAAP (Repositórios Científicos de Acesso Aberto de Portugal), two academic digital repositories of Portuguese research.

Although the review primarily focuses on Portuguese data, there were no language restrictions. Indeed, the review team includes researchers fluent in Portuguese, Spanish, English, and French. When necessary, other languages were translated using professional websites.

### 2.3. Study Selection

After completing the search, we imported all of the retrieved citations into EndNote v.21 for both automatic and manual duplicate removal. Two reviewers (AF, AP) independently screened the titles and abstracts to determine eligibility. We then retrieved potentially relevant studies in full based on the established inclusion criteria, and the reasons for exclusion were documented. Whenever necessary, disagreements were solved through discussion or with a third reviewer (SP).

### 2.4. Data Extraction

We extracted data from each included study using a predesigned data extraction form, independently completed by two reviewers (AF, AP), who had previously piloted the form to ensure consistent interpretation. We collected information on authorship, year of publication, study objectives, study design, study setting, participant details (including number, sociodemographic, and clinical characteristics), and the instruments used to measure satisfaction with care and satisfaction level. In cases of disagreement, the decision was achieved through discussion or with a third reviewer (SP).

### 2.5. Quality Assessment

We independently assessed the quality of studies using the Risk of Bias Assessment Tool for Non-Randomized Studies (RoBANS) [32], with two reviewers (AF, AP) conducting this evaluation. The assessment criteria included participant selection, confounding variables, intervention measurement, blinding of outcome assessment, completeness of outcome data, and selective outcome reporting. Each criterion was rated as having a ‘low risk of bias’, ‘high risk of bias’ or ‘unclear’ status. Whenever necessary, disagreements were solved by consensus or with a third reviewer (SP).

### 2.6. Ethical Procedures

While ethical approval was not required for this systematic review, we ensured ethical standards through a strict adherence to established methodologies and principles of health research.

## 3. Results

### 3.1. Identified Studies and Quality Appraisal

**Figure 1:**
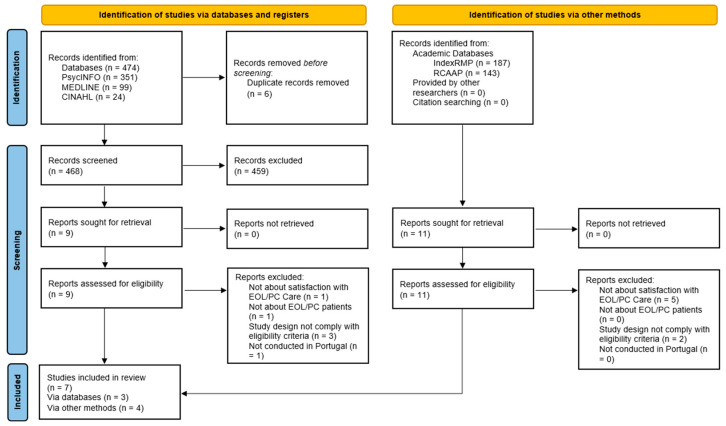
PRISMA flowchart, adapted from Page MJ, et al. [28].

Searches of electronic databases identified 468 hits after removing duplicates (Figure 1). Following the screening, 465 hits were excluded, leaving three eligible studies. Searches of gray literature yielded 330 records and added four eligible studies. No studies were found through other researchers or through citation searching. This resulted in a total of seven studies. Although one of the studies includes validation data for an instrument [33], we considered that the main objective of the study was the analysis of family members’ satisfaction of individuals receiving palliative care, and that it described important information that could not be overlooked in this review.

The quality of the included studies is summarized in Table 1. Regarding the risk of bias due to participant selection, all studies were evaluated as having a ‘low risk of bias’. Each study was also evaluated as having a ‘low risk of bias’ for confounding variables. Two studies were rated as having a ‘high risk of bias’ for intervention measurement, and one for blinding of outcome assessment. Concerning incomplete outcome data, four studies were found to have a ‘low risk of bias’ and two were classified as ‘unclear’. Selective outcome reporting was deemed ‘unclear’ in two studies and as having a ‘low risk of bias’ in four studies. Most criteria were classified as either ‘unclear’ or as having a ‘high risk of bias’ in two studies.

### 3.2. Characteristics of Studies

The studies included in this review (Table 2) were published between 2012 and 2023, detailing data from 47 patients and 758 family caregivers.

One study included patients as participants [34], while five studies focused on family caregivers [33,35,36,37,38], with one specifically involving bereaved family caregivers [39]. All of the studies focused on family caregivers’ satisfaction with care (n = 6) [33,35,36,37,38,39], including one focused on bereaved family caregivers’ satisfaction [39], except for one study that asked participants about patient satisfaction [34]. The sample size ranged from 47 [34] to 292 individuals [36]. All studies provided information on the age, sex, and education level of participants, except for one study [39], which only included information on sex.

The participants varied in age, sex, and medical conditions, reflecting a heterogeneous study population. The average age of patients was 65.38 years (range 43 to 85 years), with most being female (53.2%). Family caregivers had an average age of 51.98 years (range 20 to 87 years) and were predominantly female, married, and spouses of the patient. In most cases, their highest level of education was primary education.

The clinical characteristics of the patients were not reported in 4 studies [34,35,36,38]. However, it was possible to confirm that participants met our inclusion criteria, as they were receiving care in palliative care units. In the studies with available information [37,39], it was observed that most clinical conditions in both instances are associated with oncological diseases.

The included studies predominantly employed quantitative, descriptive, and cross-sectional designs (n = 6) [33,34,35,36,37,38]. Within this group, five studies further incorporated correlational analyses to explore relations between variables [33,34,35,36,38]. Only one study used a qualitative, exploratory, and descriptive approach, specifically drawing upon comprehensive phenomenology to understand the lived experiences of participants [39].

### 3.3. Care Setting

Although our review encompassed patients in EOL care or receiving palliative care in a variety of care settings, most of the studies (n = 5) were conducted within inpatient units [34,35,36,38,39]. Two studies explored mixed care settings, with one spanning inpatient and community settings [37] and the other outpatient and community settings [33]. Three studies focused on a single palliative care unit or team [34,35,39], while four studies focused on more than one unit or team [33,36,37,38].

Regarding geographical coverage, the studies were conducted across distinct regions of the country. Most (n = 5) were conducted in the central region of Portugal [34,35,36,38,39], while one study was conducted in the Alentejo and Lisbon regions [33]. Additionally, one study encompassed multiple areas, including both mainland Portugal and the islands [37].

### 3.4. Instruments and Level of Satisfaction with EOL Care

A variety of instruments were used across the studies to assess satisfaction with EOL care (Table 3). Regarding the instruments used to assess satisfaction with EOL care, four studies employed the Family Caregiver Satisfaction with Palliative Care (FAMCARE) scale [33,35,37,38], which is specifically designed to evaluate satisfaction from the perspective of family caregivers. The FAMCARE scale [33] yields scores ranging from 20 to 100 points; in these studies, mean scores varied between 30.84 and 77.91. The lowest mean score was observed in a sample of family caregivers from outpatient and community care settings in the Alentejo and Lisbon regions [33], while the highest score was reported in an inpatient setting in the central region of Portugal [36].

Two additional studies also focused on family caregivers, using instruments specifically developed to assess their satisfaction with care, both based on a 5-point Likert scale [35,39]. Reported satisfaction levels in these studies ranged from 96.3% [35] to 100.0% [39].

Only one study, conducted by Alves et al. [34], included patients as participants. This study utilized the Users’ Satisfaction with Nursing Care instrument (SUCEH21), which exclusively measures satisfaction with nursing care. The reported mean score was 2.42 on a scale ranging from 0 to 3. A total of 16 different variables were examined across the studies in relation to satisfaction with care, including: age [34,36], family functionality [36], gender [34,36], marital status [34,36], rurality of place of residence [36], educational level [36,38], family type [38], assuming the role of primary caregiver [38], having a family caregiver with a profession in the healthcare field [38], knowledge about palliative care [35], perception of healthcare quality [34], presence of a reference visitor [34], quality of life [33], anxiety [33], stress [33], and depression [33]. Only four variables were tested in more than one study: age [34,36], gender [34,36], marital status [34,36], and educational level [36,38]. Among these, gender and marital status do not appear to influence satisfaction with care. The influence of age and educational level on satisfaction is not consistent across the two studies.

## 4. Discussion

Although the study and assessment of satisfaction in the context of healthcare have gained significant importance in Portugal in recent years [41], the evidence regarding satisfaction with care received by adults at the EOL in this country is heterogeneous, as different populations were targeted, different instruments were used, and different variables correlated to satisfaction were studied. Although palliative care emerged in the United Kingdom in the 1960s in association with the hospice movement, in Portugal this type of care emerged linked to pioneering initiatives in the early 1990s [42]. It was only in 2004 that the national palliative care plan was introduced [43], and only in 2012 was the palliative care framework law published [44]. The first time that the level of satisfaction was mentioned as a quality indicator in the context of palliative care in Portugal was in 2013. A doctoral thesis entitled “Quality Indicators for Palliative Care Services in Portugal” used a Delphi methodology to select from 120 indicators [45]. Only in 2021, in the third strategic plan for the development of palliative care, did the evaluation of patient and family satisfaction emerge as an indicator of the evolution and continuous improvement of palliative care teams [46]. Thus, and despite international evidence pointing to the importance of satisfaction assessment in the end-of-life context, in Portugal, the national guidelines for systematically implementing this have only existed for a few years.

Although EOL care research has been evolving into more sophisticated designs, it is still complex and diverse [47]. This review shows that satisfaction with care is usually evaluated by family caregivers. In fact, in the palliative care field, it is uncommon to have patients as participants due to ethical concerns and also to difficulties in achieving adequate sample sizes, which might influence the quality of evidence produced [48]. Also, in Portugal, most research in palliative care is conducted in the academic context [49]. Deadlines regarding this research context might also influence the preference of family caregivers as participants. Even so, the importance that the academic field can have in the development of research in this area in Portugal is quite significant. The existence of various master’s programs and specialties in the central region of the country and also the implementation and development of palliative care teams might also influence the evidence highlighted in this review, as all of the studies were from the central region of Portugal, except Correia’s study, which also had data that came from different regions of mainland Portugal and Autonomous Regions [36], and Alves’s study, which also had information from the Alentejo and Lisbon regions [33]. The study involving more than one region of the country was developed in collaboration with the Portuguese Palliative Care Observatory, which is associated with a university.

In this review, all of the studies were conducted in palliative care units, teams, or services, and only two studies had information regarding patients who were not hospitalized. Previous research confirms that EOL research in the community context is scarce and that its development should be a priority in the Portuguese context [49]. Also, a systematic review characterizing EOL care in Portugal found evidence related to care provided in institutional settings, such as hospital services or palliative care units, which is consistent with these findings [50]. The fact that all of the studies included in this review were conducted in the context of palliative care confirms that satisfaction is a quality indicator associated with this type of care. Nevertheless, it is important to understand that this is neither the context nor the place where most Portuguese people die [8], creating a significant gap in information regarding satisfaction with the care received at the EOL in this country.

Although this review shows that FAMCARE is the most commonly used tool for assessing satisfaction in Portugal, this instrument was developed to measure family satisfaction with advanced cancer care in the hospital context, which is also a bias [51]. Curiously, and despite that, FAMCARE was the first referred instrument to evaluate satisfaction with care nationally, in the fourth strategic plan for the development of palliative care [46]. This might be justified by the lack of validated instruments for assessing satisfaction with EOL care. There are still few validated instruments for use in the Portuguese context in the area of EOL care [25]. Also, less than five percent of palliative care research conducted in Portugal is focused on the validation or development of assessment tools [49].

In this review, different levels of satisfaction with care were found, and the influences of 16 different variables on satisfaction with care were tested. The results regarding satisfaction with care at the end of life tend to be positive in Portugal. Recent research developed in Sweden [52] and New Zealand [53] show that overall satisfaction with care is higher in palliative care services when compared with other settings. However, we cannot affirm this is the case in Portugal, as no comparative evidence was found across different care settings. Also, there appears to be no common base regarding the study of the variables that influence satisfaction with care in Portugal. Nonetheless, it was found that the majority of the tested variables are related to personal characteristics of the patient or the caregiver/family caregiver. Only two variables are directly related to the care received: perception of healthcare quality and the presence of a reference visitor. No evidence was found regarding the study of variables related to the patient’s trajectory, location of care provision, or place of death. This seems to be consistent with the results found in a systematic review that aimed to synthesize the scientific evidence on the factors that influence the assessment of customer satisfaction with PC and identify the instruments used for its assessment [26].

The few studies included in this systematic review, in such an important theme as satisfaction with care, seem to highlight the importance of defining national research priorities in the areas of end-of-life care and palliative care. It also seems necessary to define nationally a validated tool for all settings that allows the evaluation of satisfaction with end-of-life care. It also appears necessary to assess the influence that variables directly related to the care received can have on satisfaction, as these are the ones that can be the focus of analysis and continuous improvement by the teams, regardless of their context.

## 5. Conclusions

We believe this study provided baseline evidence regarding the satisfaction with care received by adult individuals at the EOL in Portugal. This review suggests that satisfaction with the care received by adults at the end of life tends to be positive. However, we recognize that definitive conclusions cannot be drawn, as the number of included studies is limited and they all originate from the palliative care context. As a result, we were unable to compare outcomes across different settings within the Portuguese context.

Although a rigorous methodology was employed, some limitations of this review must be acknowledged. The screening phase during study selection may have been influenced by the quality of the papers’ abstracts. We also recognize that the included studies present some risk of bias, as identified during the quality assessment.

Nonetheless, this review is the first attempt to determine the Portuguese reality regarding the characterization of satisfaction with end-of-life care received by adults, which may also raise awareness among healthcare professionals about the importance of evaluating satisfaction with end-of-life care, both as a means to promote respect for human dignity and as a tool to help reduce the gap between patients’ and families’ expectations and the care provided. From a research perspective, we conclude there is a clear need to develop and validate methodological instruments to assess satisfaction that are applicable across all care settings. Additionally, further studies are needed to understand the levels of satisfaction with end-of-life care across the entire country and in all contexts.

At the policy level, we believe it is necessary to recognize the role that satisfaction assessment at the end of life can play in the continuous improvement of healthcare organization and delivery. Therefore, national recommendations and guidelines should be made within the Portuguese healthcare system, which should not only include the evaluation of satisfaction but also gather information about the patient’s personal characteristics, illness, and trajectory in using social and healthcare resources. These aspects are essential to ensure comparability and to guarantee the development of measures related to the continuous improvement of EOL healthcare quality throughout Portugal.

## Figures and Tables

**Table 1:** Quality of studies.

Risk of Bias	Almeida, 2012 [33]	Alves, 2015 [34]	Fontes, 2015 [35]	Bica et al., 2016 [36]	Correia, 2018 [37]	Ribeiro et al., 2020 [38]	Soares et al., 2023 [39]
Selection of participants	○	○	○	○	○	○	○
Confounding variables	○	○	○	○	○	○	○
Intervention measurement	□	○	○	○	□	○	○
Blinding of outcome assessment	○	○	○	○	○	○	□
Incomplete outcome data	○	○	X	○	○	○	X
Selective outcome reporting	○	○	X	○	○	○	X

Footnote: ○: Low risk of bias □: High risk of bias X: Unclear.

**Table 2:** Included studies: study objectives and designs, setting, patients’ clinical characteristics, participants (type and sociodemographic characteristics) and instruments.

Authors, Year of Publication	Study Objectives	Study Design	Setting (Type of Service|Region of Country)	Patients’ Clinical Characteristics	Participants (Type, Number)	Participants’ Sociodemographic Characteristics	Instruments
Almeida, 2012 [33]	To describe the family caregivers’ satisfaction regarding the care provided to patients in PC	Quantitative, descriptive, cross-sectional, correlational study	Outpatient and community care (PC Unit and Community PC Support Team)|Alentejo and Lisbon Region	No specific information provided; Patients were under PC treatment	Family caregivers (n = 103) of outpatients (n = 78) and community care patients (n = 25)	Age M = 58.40 years ± SD 15.22 78.6% female 86.4% married 61.2% primary education 51.5% were spouses	FAMCARE (Portuguese version) ^a^
Alves, 2015 [34]	To understand the patterns of satisfaction with healthcare and nursing care as perceived by people at the EOL	Quantitative, descriptive, cross-sectional, correlational study	Inpatient (PC Unit)|Central Region of Portugal	No specific information provided; patients were hospitalized for more than a week in the PC unit	Patients (n = 47) hospitalized in a PC unit	Age M = 65.38 years ± SD 9.69 53.2% female 48.9% married 42.6% primary education	SUCEH21 (Portuguese version) ^b^
Fontes, 2015 [35]	To assess the level of satisfaction of the families of patients hospitalized in a Palliative Medicine Service	Quantitative, descriptive, cross-sectional, correlational study	Inpatient (Palliative Medicine Service)|Central Region of Portugal	No specific information provided; Patients had a life-threatening condition and were hospitalized in the Palliative Medicine Service	Family caregivers (n = 53) providing direct care to patients hospitalized in the Palliative Medicine Service	Age M = 55.15 years ± SD 14.02 66.0% female 28.3% secondary education 60.4% were sons/daughters	Own-designed instrument (Assessment of satisfaction with a 5-point Likert scale question)
Bica et al., 2016 [36]	To identify the socioeconomic variables that affect the satisfaction of families of patients in PC	Quantitative, descriptive, cross-sectional study	Inpatient (PC Units)|Central Region of Portugal	No specific information provided; patients were hospitalized in the PC unit	150 Family caregivers (n = 150) of PC patients hospitalized in PC Units	73.3% female (Age M = 35.45 years ± SD 15.05; 57.3% single) 26.7% male (Age M = 41.30 years ± SD 17.69; 52.5% married) 39.3% secondary education	FAMCARE (Portuguese version) ^a^
Correia, 2018 [37]	To characterize the caregiver of the palliative patient, assess their satisfaction, and identify the factors that influence it	Quantitative, descriptive, cross-sectional study	Inpatient and community care (2 PC Units, 1 Intrahospital PC Support Team and 3 Community Palliative Care Support Teams)|Mainland Portugal and Autonomous Regions	84.8% oncology disease 14.1% non-oncology disease 1.1% mixed disease	Family caregivers (n = 292) of patients supported by PC teams/units	Age M = 57.49 years ± SD 13.61 68.5% female 79.4% married 62.4% primary education 40.9% were spouses	FAMCARE (Portuguese version) ^a^
Ribeiro et al., 2020 [38]	To evaluate the degree of satisfaction of family caregivers of patients hospitalized in PC regarding the care provided and to analyze their relationship with sociodemographic variables	Quantitative, descriptive, cross-sectional, correlational study	Inpatient (3 PC Units)|Central Region of Portugal	No specific information provided; patients were hospitalized in 1 of 3 PC units	Family caregivers (n = 96) of patients hospitalized in PC Units	Age M = 49.44 years ± SD 14.90 66.7% female 64.6% married 49.0% primary education 41.7% were sons/daughters	FAMCARE (Portuguese version) ^a^
Soares et al., 2023 [39]	To assess the degree of satisfaction of relatives of patients in the PC unit.	Qualitative Exploratory and Descriptive Study Based on Comprehensive Phenomenology	Inpatient (PC Unit)|Central Region of Portugal	92.0% oncology disease	Bereaved Family caregivers (n = 64) of patients hospitalized in the PC unit	48.44% female 43.75% were sons/daughters	Own-designed instrument (Assessment of satisfaction based on the information provided by the family caregiver through a telephonic interview, and subsequently converted into a 5-point Likert scale)

Footnote: M—mean; PC—palliative care; SD—standard deviation; ^a^ FAMCARE Portuguese Version [33]; ^b^ SUCEH21 Portuguese Version [40].

**Table 3:** Included studies: variables that were tested as having an influence on satisfaction with care and their statistical significance.

Authors, Year of Publication	Satisfaction Evaluator	Instrument (Score Range)	Satisfaction with Care (Score)	Studied Variables	Variables with Statistical Significance	Interpretation of Statistical Significance
Almeida, 2012 [33]	Family caregivers	FAMCARE (20 to 100 points)	30.84 points	- quality of life - anxiety - depression - stress	No statistically significant relations were found	Not applicable
Alves, 2015 [34]	Patients	SUCEH21 (0 to 3 points)	2.42 points	- age - gender - marital status - perception of healthcare quality - presence of a reference visitor	Perception of healthcare quality and the presence of a reference visitor were found to have a statistically significant relation	People at the EOL who are more satisfied with nursing care tend to provide a better evaluation of healthcare quality. People at the EOL who had a reference visitor tend to show higher satisfaction with nursing care.
Fontes, 2015 [35]	Family caregivers	Own-designed instrument	62.3% of the family caregivers were very satisfied with the care provided, 34.0% were satisfied, and the remaining were undecided or dissatisfied	- knowledge about PC	No statistically significant relation was found	Not applicable
Bica et al., 2016 [36]	Family caregivers	FAMCARE (20 to 100 points)	77.91 points	- age - gender - marital status - rurality of the place of residence - educational level - family functionality	Age and family functionality were found to have a statistically significant relation	The less satisfied family members are those who are 26 years old or younger. Participants with high family functionality show a higher level of satisfaction in all dimensions of the scale.
Correia, 2018 [37]	Family caregivers	FAMCARE (20 to 100 points)	76.68 points	No variables tested	Not applicable	Not applicable
Ribeiro et al., 2020 [38]	Family caregivers	FAMCARE (20 to 100 points)	40.50 points	- education level - family type - assuming the role of primary caregiver - having a reference family caregiver with a profession in the healthcare field	All of them were found to have a statistically significant relation.	Single-person families are more dissatisfied with healthcare, while single-parent families are more satisfied. A lower level of education is associated with a higher degree of satisfaction. Assuming the role of the primary caregiver positively influences satisfaction with the care. Reference family caregivers with professions related to the healthcare field negatively influence the degree of satisfaction with care in the dimensions of physical and psychosocial care.
Soares et al., 2023 [39]	Bereaved Family caregivers	Own-designed instrument	92.19% of bereaved relatives reported being completely satisfied with the care received, and 7.81% reported being very satisfied	No variables tested	Not applicable	Not applicable

## Data Availability

The original contributions presented in this study are included in the article. Further inquiries can be directed to the corresponding author.

## Public Involvement Statement

There was no public involvement in any aspect of this research.

## Guidelines and Standards Statement

This manuscript was drafted using the Joanna Briggs Institute methodology for systematic reviews, and it was reported using the Preferred Reporting Items for Systematic Reviews and Meta-analyses guidelines.

## Abbreviations

The following abbreviations are used in this manuscript:

CIBB	Centre for Innovative Biomedicine and Biotechnology
DOAJ	Directory of open access journals
EOL	End-of-Life
FAMCARE	Family Caregiver Satisfaction with Palliative Care
IndexRMP	Índex de Revistas Médicas Portuguesas
M	Mean
MDPI	Multidisciplinary Digital Publishing Institute
PC	Palliative Care
PRISMA	Preferred Reporting Items for Systematic Reviews and Meta-analyses
RCAAP	Repositórios Científicos de Acesso Aberto de Portugal
RCTs	Randomized Controlled Trials
RoBANS	Risk of Bias Assessment Tool for Non-Randomized Studies
SD	Standard Deviation
SUCEH21	Users’ Satisfaction with Nursing Care instrument

## Appendix A

**Table A1:** Search Strategy.

Database	Combined Boolean (Controlled and Free Text)
CINAHL (EBSCOhost)	((MH “Palliative Care” OR MH “Terminal Care”) OR (“palliative care” OR “end-of-life” OR “hospice care”)) AND ((MH “Patient Satisfaction” OR MH “Family Satisfaction”) OR (“satisfaction” OR “patient satisfaction” OR “care satisfaction”)) AND ((MH “Portugal”) OR (“Portugal”))
MEDLINE (via PubMed/Ovid)	((“Palliative Care” [Mesh] OR “Terminal Care” [Mesh]) OR (“palliative care” OR “end-of-life” OR “hospice care”)) AND ((“Patient Satisfaction” [Mesh]) OR (“satisfaction” OR “patient satisfaction” OR “care satisfaction”)) AND ((“Portugal” [Mesh]) OR (“Portugal”))
PsycINFO (via Ovid)	((DE “Palliative Care” OR DE “Terminal Care”) OR (“palliative care” OR “end-of-life” OR “hospice care”)) AND ((DE “Client Satisfaction” OR DE “Patient Attitudes”) OR (“satisfaction” OR “client satisfaction” OR “patient satisfaction”)) AND (“Portugal”)
